# A shot to the gut: appendicitis triggered by retained buckshot

**DOI:** 10.1093/jscr/rjaf423

**Published:** 2025-06-18

**Authors:** Georges Kaoukabani, Roberto Alva-Ruiz, Omair Shariq, Joseph T Carroll, John M Zietlow

**Affiliations:** Department of Surgery, Mayo Clinic, 200 1st St. SW, Rochester, MN 55905, United States; Department of Surgery, Mayo Clinic, 200 1st St. SW, Rochester, MN 55905, United States; Department of Surgery, Mayo Clinic, 200 1st St. SW, Rochester, MN 55905, United States; Department of Surgery, Hennepin Healthcare, 701 Park Ave. S, Minneapolis, MN 55415, United States; Department of Surgery, Mayo Clinic, 200 1st St. SW, Rochester, MN 55905, United States

**Keywords:** appendicitis, foreign object, acute care surgery, buckshot

## Abstract

Acute appendicitis is a common surgical emergency, most often caused by luminal obstruction from fecaliths. Rarely, foreign body ingestion can lead to appendicitis, particularly when small objects like lead shot become lodged in the appendix. We present the case of a 30-year-old man with chronic intermittent right lower quadrant pain who presented with an acute exacerbation. Imaging revealed two metallic densities in the appendix without overt signs of inflammation. He underwent laparoscopic appendectomy, which confirmed the presence of two retained lead pellets and histologic evidence of acute appendicitis. The patient had a history of frequent game meat consumption. This case underscores a rare but important etiology of appendicitis and the potential for systemic lead exposure from retained foreign bodies. In patients with relevant social histories and suggestive imaging, clinicians should consider this diagnosis and proceed with timely surgical management to prevent complications.

## Introduction

Acute appendicitis remains one of the most prevalent surgical emergencies worldwide [1]. Acute appendicitis is primarily triggered by obstruction of the appendiceal lumen, most commonly due to fecaliths, which leads to an overgrowth of the naturally occurring aerobic and anaerobic bacteria [2]. While rare, instances of ingested foreign bodies dislodged into the appendix have been reported in the literature [3]. We present one such case below.

## Case report

**Figure 1:**
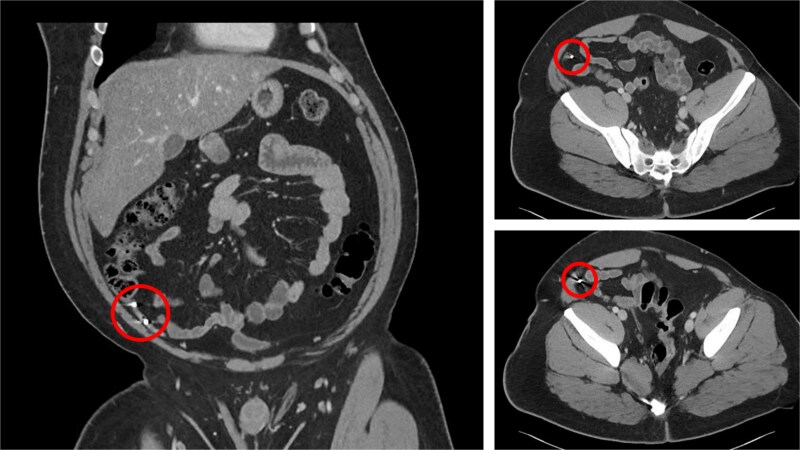
CT of the abdomen of the patient.

**Figure 2:**
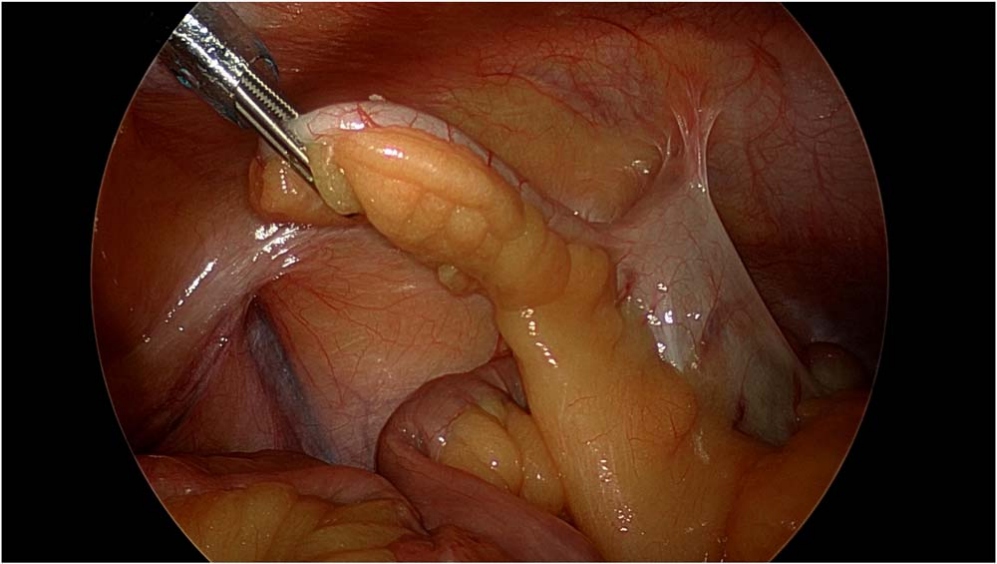
Laparoscopic view of the inflamed appendix.

**Figure 3:**
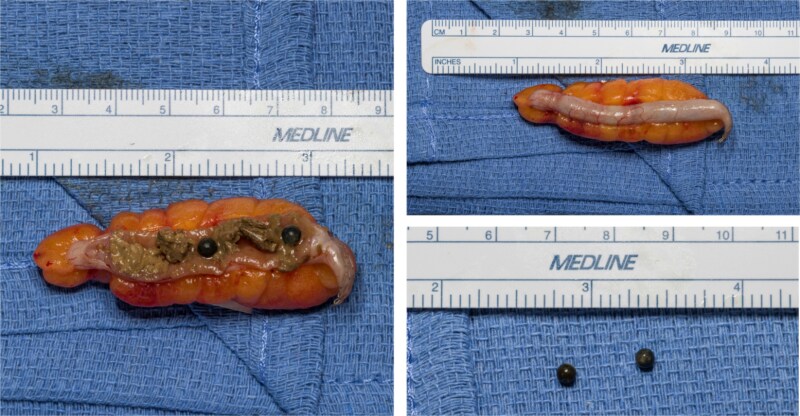
Appendix at bedside with retained buckshot.

A 30-year-old gentleman with a medical history relevant for asthma and no previous abdominal surgeries presented to the emergency department (ED) at Saint Mary’s Hospital, Rochester, MN, USA, for acute abdominal pain. He described a longstanding history of intermittent right lower quadrant pain, amounting to more than a year and a half. His pain worsened the day prior to his presentation to the ED while attempting to have a bowel movement. On the physical exam, he endorsed a focal tenderness to the right lower quadrant with deep palpation. His laboratory values did not show any derangements, with a hemoglobin level of 15.4 g/dl and a total white blood cell count of 8.6 × 10^9^/l. A computed tomography (CT) scan of the abdomen and pelvis was performed and demonstrated the presence of two rounded, identical metallic bodies in the appendix, measuring up to 5 mm, with no signs of acute appendicitis (Fig. 1). Upon further investigation, it was discovered that the patient was an avid hunter who regularly consumed his game. He was subsequently consented for a laparoscopic appendectomy and taken to the operating room. The abdomen was entered in the standard fashion, and three laparoscopic ports were placed. The appendix was quickly identified in its normal anatomical position, and mild inflammation was noted (Fig. 2). A 45 mm tan Endo GIA stapler was utilized to staple across the base of the appendix, which was then removed via an Endo Catch bag through a 10 mm port. It was opened on the back table, and two small pellets were identified to be consistent with preoperative imaging (Fig. 3). The specimen was sent to the pathology laboratory, which confirmed the diagnosis of acute appendicitis. The patient tolerated the procedure well and was eventually discharged home on postoperative day one.

## Discussion

Foreign bodies have been reported in the literature as a potential cause of acute appendicitis, with few reports identifying ingested lead shots as an unusual etiology. These metallic pellets found in game meat can migrate through the gastrointestinal tract and occasionally lodge in the appendix, considering their small size and round shape, thus perfectly resembling fecaliths.

The presence of lead adds another layer of clinical concern. Its toxic properties can contribute to localized inflammation, tissue damage, or even systemic toxicity if retained. Of particular concern is the risk of chronic lead poisoning, especially in pediatric patients, due to slow mucosal absorption over time [4]. This underscores the importance of prompt identification and removal of such foreign bodies.

Although rare, appendicitis caused by lead shot ingestion should be suspected in the appropriate social background and imaging showing metallic bodies in the appendix. Prompt surgical intervention is vital not only to treat appendicitis but also to prevent systemic lead exposure, underlining the importance of removing such foreign bodies.

## References

[1] Lotfollahzadeh S, Lopez RA, Deppen JG . Appendicitis. StatPearls. Treasure Island (FL): StatPearls Publishing. 2024. https://www.ncbi.nlm.nih.gov/books/NBK493193/# (16 April 2025, date last accessed).

[2] Bhangu A, Søreide K, Di Saverio S, et al. Acute appendicitis: modern understanding of pathogenesis, diagnosis, and management. Lancet 2015;386:1278–87. 10.1016/S0140-6736(15)00275-5 26460662

[3] Elmansi Abdalla HE, Nour HM, Qasim M, et al. Appendiceal foreign bodies in adults: a systematic review of case reports. Cureus 2023;15:e40133. 10.7759/cureus.40133 37425596 PMC10329456

[4] Durback LF, Wedin GP, Seidler DE. Management of lead foreign body ingestion. J Toxicol Clin Toxicol 1989;27:173–82. 10.3109/15563658909038581 2681811

